# Racial Differences in Perceptions of Air Pollution Health Risk: Does Environmental Exposure Matter?

**DOI:** 10.3390/ijerph14020116

**Published:** 2017-01-25

**Authors:** Jayajit Chakraborty, Timothy W. Collins, Sara E. Grineski, Alejandra Maldonado

**Affiliations:** Department of Sociology and Anthropology, University of Texas at El Paso, El Paso, TX 79968, USA; twcollins@utep.edu (T.W.C.); segrineski@utep.edu (S.E.G.); amaldonado7@miners.utep.edu (A.M.)

**Keywords:** risk perception, air pollution, cancer risk, race/ethnicity, Houston

## Abstract

This article extends environmental risk perception research by exploring how potential health risk from exposure to industrial and vehicular air pollutants, as well as other contextual and socio-demographic factors, influence racial/ethnic differences in air pollution health risk perception. Our study site is the Greater Houston metropolitan area, Texas, USA—a racially/ethnically diverse area facing high levels of exposure to pollutants from both industrial and transportation sources. We integrate primary household-level survey data with estimates of excess cancer risk from ambient exposure to industrial and on-road mobile source emissions of air toxics obtained from the U.S. Environmental Protection Agency. Statistical analysis is based on multivariate generalized estimation equation models which account for geographic clustering of surveyed households. Our results reveal significantly higher risk perceptions for non-Hispanic Black residents and those exposed to greater cancer risk from industrial pollutants, and also indicate that gender influences the relationship between race/ethnicity and air pollution risk perception. These findings highlight the need to incorporate measures of environmental health risk exposure in future analysis of social disparities in risk perception.

## 1. Introduction

Under the rubric of environmental justice (EJ) research, a wide range of quantitative case studies have found neighborhoods with higher proportions of racial/ethnic minority and socioeconomically disadvantaged residents to be disproportionately exposed to various environmental risks in the U.S. [1,2,3,4]. EJ advocates and scholars have emphasized the need to shift the nature of inquiry in the area of environmental concern from environmental protection to how people and communities are directly affected, in the places where they live, by environmental hazards emanating from industry, transportation, and hazardous waste facilities [5,6,7]. However, few studies have examined how concerns or perceptions associated with environmental hazards such as air pollution are influenced by ambient exposure to toxic chemicals from local sources, especially for racial/ethnic minorities who are likely to face greater exposure to such environmental health risks. 

A number of prior studies suggest that racial/ethnic minorities demonstrate higher levels of environmental concern than Whites, especially when such concerns are framed in terms of environmental health risk [8,9,10,11,12]. Even when framed in more general terms, African Americans have been found to have at least as much, if not more, concern for the environment than Whites [13,14,15]. Two different explanations have been proposed related to how racial/ethnic differences in risk perception are influenced by local exposure to environmental risk [16]. The literature on environmental social movements indicates that people exposed to higher levels of air pollution risk tend to have heightened risk perceptions and are more likely to mobilize based on perceived injustices [5,17,18,19,20]. This suggests that minority residents may exhibit higher levels of risk perception compared to Whites, due to their actual exposure to environmental risks. A separate strand of qualitative research focused on the formation of collective perceptions of risk has emphasized social psychological processes that tend to dampen risk perceptions among residents who are exposed to serious toxic health threats [21,22]. In contrast to the social movements literature, qualitative studies on collective risk perception suggest that residents who experience greater exposure to pollution often exhibit unrealistically lower perceptions of environmental risk. Consequently, the collective attenuation of risk perceptions among minority communities exposed to high environmental risk may reinforce the distributional injustices these residents confront [16]. This supports the expectation that minority residents might exhibit lower levels of risk perception compared to Whites, when controlling for their actual risk exposures. Given this ambiguity and dichotomy in the literature, more empirical research is necessary to determine how everyday exposure to chronic hazards such as air pollution affects the relationship between race/ethnicity and environmental risk perception.

Empirical research on racial/ethnic differences in environmental risk perception is characterized by several additional limitations. A recent review of this literature [7] identified two specific weaknesses: (a) excessive emphasis on dichotomous White–Black or White–non-White differences, neglecting growing diversity in the U.S. population, and in particular, individuals of Hispanic/Latino origin; and (b) failure to adequately control for gender, income, education, and other contextual factors that should be included in an analysis of racial/ethnic correlates. Gender is a particularly relevant variable because it has been found to significantly influence relationships between race/ethnicity and environmental risk perception [23,24]. While previous research suggests that White males exhibit lower risk perceptions and greater willingness to accept risks compared to women and non-White men [25,26,27], few studies have examined how exposure to industrial or vehicular air pollution can bias these interrelationships. Another limitation that has received relatively less attention focuses on the statistical approach used to evaluate the relationship between environmental risk perception and racial/ethnic variables. A majority of studies have relied on multivariate regression models based on the least squares regression approach without adequately verifying whether the statistical assumptions of this approach have been met by their survey data. Statistical associations between perceived environmental risk and racial/ethnic status, for example, could be spurious if these variables tend to cluster together or are influenced by values of proximate observations (e.g., perceptions of neighboring residents), as demonstrated by recent quantitative studies on social disparities in the distribution of environmental risks [28,29,30]. Regression techniques that control for geographic clustering are necessary to clarify whether racial/ethnic and other explanatory variables are truly robust in multivariate analysis of environmental risk perception. 

This article seeks to address the aforementioned limitations of previous work and extend environmental risk perception research by exploring how the spatial pattern of exposure to industrial and vehicular air pollutants and other contextual factors influence racial/ethnic differences in perceptions of air pollution health risks. The study site for this research is the Greater Houston metropolitan area, Texas, USA—a racially/ethnically diverse urban area with numerous neighborhoods facing high levels of exposure to toxic air emissions from industrial and transportation sources. Our specific objectives are to examine: (a) the relationship between race/ethnicity and air pollution health risk perception at the individual/household level; and (b) how this relationship changes when we consider exposure to both industrial and vehicular air pollution, as well as other contextual and socio-demographic variables such as gender, age, home ownership, socioeconomic status, and previous experience with air pollution that have been found to influence perceived environmental risks in previous research; and (c) if the relationship between race/ethnicity and air pollution risk perception is conditional on gender, after controlling for other contextual and socio-demographic characteristics. Our study integrates primary household-level survey data with modeled estimates of potential cancer risk from exposure to industrial toxic releases and on-road mobile source emissions of hazardous air pollutants that were obtained from the U.S. Environmental Protection Agency (USEPA). In order to analyze racial/ethnic differences in environmental risk perception, we use generalized estimation equation (GEE) models, which account for geographic clustering of surveyed households in our study area and provide more statistically valid inferences regarding the environmental and social determinants of air pollution health risk perception.

## 2. Materials and Methods

### 2.1. Study Area

**Figure 1:**
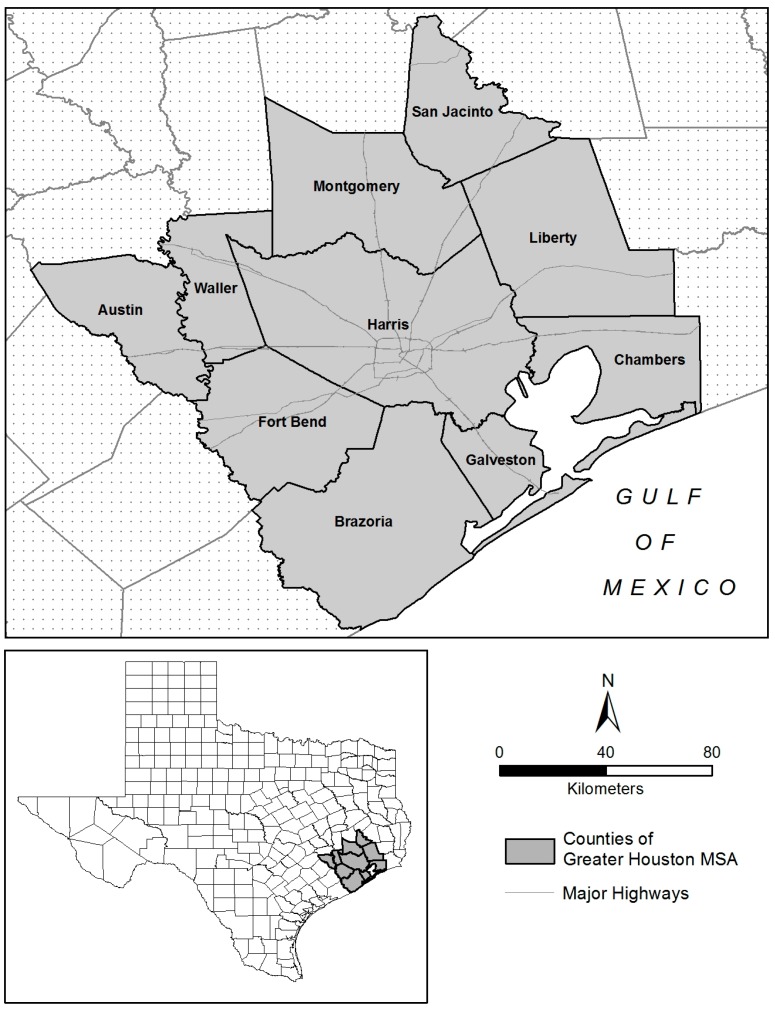
Location of the Greater Houston Metropolitan Statistical Area (MSA), 2011, Texas, USA.

The Greater Houston Metropolitan Statistical Area (MSA) comprises ten counties in southeastern Texas, as depicted in Figure 1. This MSA is bordered on the southeast by the Gulf of Mexico and is intersected by several major interstate highways. With a total population of about 5.9 million (2010), it is the largest MSA in Texas and the sixth largest MSA in the U.S. The three most populous cities in the MSA include Houston, the Woodlands, and Sugar Land. According to the 2010 U.S. Census, non-Hispanic Whites account for about 39.7% of the MSA population, with Hispanics (35.3%) and non-Hispanic Blacks (16.8%) representing the largest minority groups.

The Greater Houston MSA is a suitable study area for this research because of its racial/ethnic diversity and ambient air pollution problems. Airborne emissions from numerous point and on-road sources have contributed to elevated levels of chronic exposure to various types of air pollutants in this MSA. A report compiled by the *Mayor’s Task Force on the Health Effects of Air Pollution* concluded that air pollution levels in this metropolitan area are considered to be unacceptable by knowledgeable experts and the general public, and are likely to cause air pollution-related health effects for residents [31].

Both industrial and transportation-related air pollution have emerged as significant health threats for residents of Greater Houston. Chronic exposure to industrial air pollutants is of particular concern in this MSA, based on the nature and quantity of toxic chemical releases resulting from petrochemical industrial activities. This MSA is one of the world’s largest manufacturing centers for petrochemicals and home to the largest petrochemical complex in the U.S., in addition to hosting two of the four largest refineries in the nation [31]. The petrochemical industry is served by more than 400 chemical plants, the majority of which are located along the Houston Ship Channel that flows 50 miles inland from the Gulf of Mexico [32]. The urban development of Greater Houston has prioritized minimizing costs for potential investors to locate in the MSA, which has simultaneously created less than ideal living conditions, especially for minority communities that are located near the ship channel [33]. On-road emissions from motorized vehicles have also emerged as a serious public health concern in Greater Houston, as daily traffic volumes have increased substantially in the last two decades. In 2005, the Greater Houston MSA was ranked first among all U.S. metropolitan areas in terms of both daily vehicle miles traveled (VMT) per capita and increase in per capita VMT between 1992 and 2005 [34]. Tailpipe emissions from cars, trucks and buses were also identified by the Mayor’s Task Force as one of the most important source categories for air pollution health risks in this urban area [31]. 

Although recent EJ studies have reported racial/ethnic and socioeconomic inequities in the distribution of exposure to toxic air pollutants in Greater Houston [16,35] previous research has not investigated how racial/ethnic differences in risk perception are potentially influenced by ambient exposure to emissions from both industrial and transportation sources. The Collins et al. [16] study represents the first attempt to directly incorporate risk perception as an explanatory variable in EJ analysis, but found a statistically non-significant relationship between survey participants’ air pollution health risk perceptions and their exposure to air toxics and cancer risk. The only risk perception study that focused specifically on outdoor air pollution in the Houston metropolitan area [36] concluded that perceptions of local air quality were not consistent with actual readings from air monitoring stations, but were shaped by other factors such as access to information and socioeconomic characteristics. While this study did not specifically focus on racial/ethnic disparities or examine exposure to health risks from industrial or vehicular air pollutants, air quality perception differences between non-White (treated as a single group) and White residents were found to be non-significant.

### 2.2. Sampling and Survey

The data used to construct the dependent and a majority of independent variables for this study was derived from an Institutional Review Board (IRB)-approved cross-sectional telephone survey of randomly selected adults in the Greater Houston MSA that was conducted from 28 June to 1 August 2012. The human subjects research protocol (FWA: 00001224; internal IRB reference No.: 261207-4) was approved by the University of Texas at El Paso IRB on 18 May 2012. The telephone interviews were conducted by trained, English-Spanish bilingual interviewers employed by a firm with expertise in survey research in this metropolitan area. Each survey lasted approximately 30 min and incentives of $10 in cash were provided to all participants who completed the survey. Additional information regarding the structured survey instrument and related details are available in Collins et al. [16].

**Figure 2:**
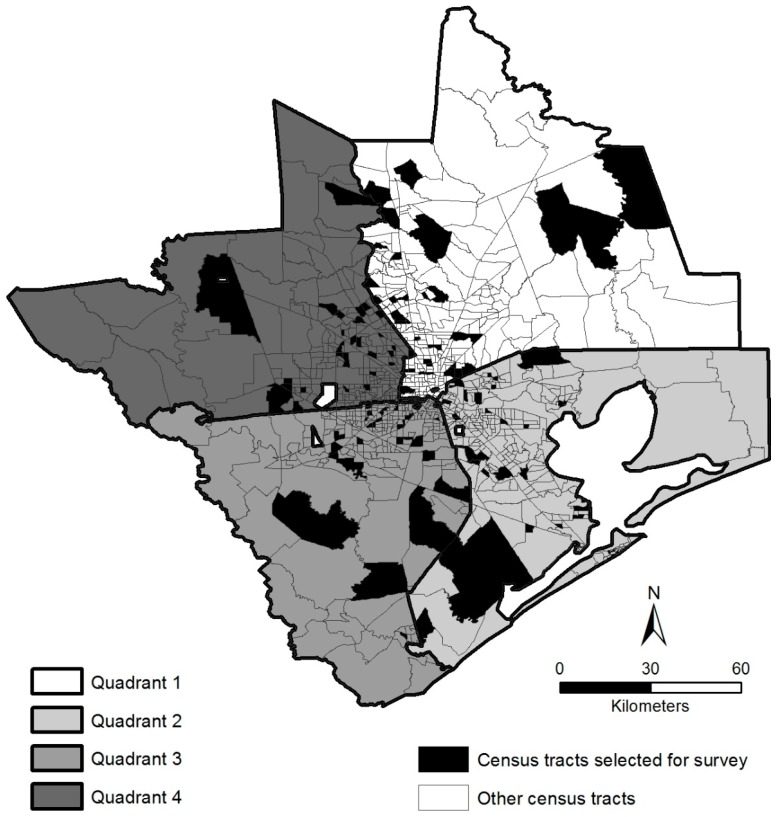
Census tracts selected for household level telephone survey in the Greater Houston MSA, 2012.

A multi-stage sampling approach was utilized to ensure that our sample was drawn randomly using probability methods, as well as being spatially and socially representative of the Greater Houston MSA. The approach involved stratifying and randomly selecting a subset of census tracts across four geographic quadrants of our study area (stage 1), and then completing telephone surveys with randomly selected residents in each selected tract (stage 2). To select the census tracts for stage 1, tracts with each quadrant were stratified into quintiles based on an index created from two census variables representing race/ethnicity and socioeconomic status: percentage of non-Hispanic whites, and median household income. Within each quintile for each quadrant, we randomly chose six tracts, with a total of 30 selected tracts per quadrant. Figure 2 shows the resulting random stratified sample of 120 census tracts. For stage 2, telephone interviews were completed with at least five randomly-selected householders in each of 120 randomly selected tracts in the Greater Houston MSA, for a targeted total of 600 completed telephone surveys. 

For sampling at the household level, we utilized listed residential landline telephone numbers because of the detailed geographic resolution necessary for our analysis and to connect each survey respondent to a home address within our selected census tracts. Although landline-only surveys might disproportionately exclude people known to have high rates of cell-only usage (e.g., Hispanic residents) from sampling frames, research indicates that this does not necessarily produce more biased estimates for those groups [37,38]. The overall response rate for the survey was 33%, which is comparable to rates reported in recent published studies that relied on random digit dialing surveys [39]. Our sampling design yielded a generally representative sample with respect to the adult population (aged 18 or more years) of the Greater Houston MSA, especially in terms of racial/ethnic characteristics of the respondents [16]. We used the address-matching capabilities of ArcGIS 10 software and Google Earth to geocode the locations of all survey respondents to the street network of the Greater Houston MSA, based on their home addresses. This study includes 586 households that were successfully geocoded and for which complete survey data are available for our dependent variable and most independent variables.

### 2.3. Measures

#### 2.3.1. Dependent Variable

Air pollution health risk perception (APHRP) was measured at the individual level by combining responses to two specific survey questions that relied on Likert-scale items to evaluate respondents’ perceptions: (1) How much of a problem do you think air pollution is in this urban area (1 = not a problem at all, to 5 = a very serious problem; mean = 3.60 and standard deviation = 1.08); and (2) How concerned are you about the possibility of air pollution causing health problems to you or members of your household (1 = not concerned, to 5 = extremely concerned; mean = 3.25 and standard deviation = 1.34). Responses to these two questions were combined to construct a two-item factor (Cronbach’s alpha = 0.77) using principal components analysis that represented the dependent variable for our statistical analysis. The use of these two survey variables allowed us to assess respondents’ perceptions regarding the seriousness of air pollution problems in the Greater Houston MSA, as well as their levels of concerns regarding potential health impacts in their own homes, thus incorporating both the local and household dimensions of the issue [40].

#### 2.3.2. Explanatory Variables: Air Pollution Health Risk

We first used secondary data sources to construct two explanatory variables that examine potential health risks associated with ambient exposure to industrial and vehicular air pollutants, respectively. To measure risk from industrial pollution sources, we used the USEPA’s Risk Screening Environmental Indicators (RSEI) model for 2011. Nationally, the RSEI model includes over 400 chemicals from more than 25,000 facilities in the manufacturing, mining, power generation, waste-management and chemical-management sectors that are required to report their chemical releases to the Toxics Release Inventory (TRI), established under the Emergency Planning and Community Right-to-Know Act in 1986. The RSEI includes a fate-and-transport model that uses information on stack heights, exit gas velocities, wind patterns, and chemical decay rates to estimate ambient concentrations of the toxic releases for 11,289 grid cells, each 810 m by 810 m, around each TRI facility. Chemicals are weighted in the RSEI by toxicity, defined in the case of air releases as chronic human health effects from inhalation exposure [41]. Combining the toxicity-weighted ambient concentrations with census data on the size and age–sex composition of populations residing in each grid cell, the RSEI calculates a risk-screening indicator for each facility, aggregated across the grid cells impacted by its releases. While the RSEI public release data only provides facility-specific hazard and risk scores for each facility, disaggregated information on individual grid cells impacted by the toxic air releases from TRI facilities is available in the RSEI Geographic Microdata (RSEI-GM). The RSEI-GM raw data provides ambient concentrations and risk estimates for each 810 m by 810 m grid cell in the continental U.S., with each cell value aggregating information from all TRI facilities whose air releases impact that location. Although the disaggregated data are not released publicly due to their large size and complexity, the USEPA has made the RSEI-GM database available to the research community. Recent EJ studies have used RSEI-GM to analyze various social disparities in industrial chemical releases [42,43,44]. 

For this study, our industrial air pollution risk variable is based on estimates of excess cancer risk extracted from the RSEI-GM database for 2011. Since our analysis is not chemical specific, we used the aggregated sum of excess cancer risk across all chemicals at the grid cell-level that results in a total excess cancer risk estimate for each 810 m by 810 m grid cell. The cancer risk scores obtained for the RSEI-GM grid cells in the Greater Houston MSA were spatially allocated to our sample of 586 surveyed households. Each geocoded survey respondent’s cancer risk from industrial air pollutants thus equals the total excess cancer score of the RSEI-GM grid cell in which their home address is located. 

To measure health risks from ambient exposure to vehicular sources of air pollutants, we used the U.S. EPA’s National-Scale Air Toxics Assessment (NATA), which has emerged as an important database for estimating health risks associated with the inhalation of hazardous air pollutants (HAPs), as well as the most reliable data source for spatially explicit characterization of HAP risk exposure in U.S. urban areas [16,45,46,47]. HAPs are also known as non-criteria air pollutants and include 188 specific substances identified by in the Clean Air Act Amendments of 1990 that are known to or suspected of causing cancer and other serious health problems [48]. Our study utilizes the 2005 NATA, which was released in 2011. Although the 2005 NATA data is available online for census tracts, we were able to acquire census block level estimates directly from the USEPA. This finer spatial resolution allowed a more accurate assessment of potential health risk from HAP exposure faced by survey respondents at their home locations, compared to tract level NATA data that has been utilized to analyze social inequities in the distribution of HAPs.

To represent our independent variable for vehicular air pollution risk, we use values of estimated lifetime cancer risk from inhalation exposure to on-road mobile sources of HAPs associated with each participating household, based on the census block within which the household resided at the time the telephone survey was conducted. The on-road mobile source category includes motorized vehicles that typically operate on public roadways and comprises passenger cars, motorcycles, minivans, sport-utility vehicles, trucks, and buses [49]. Lifetime cancer risks in the 2005 NATA are derived using unit risk estimates (URE), an upper bound estimate of an individual's probability of developing cancer over a lifetime of exposure to a concentration of one microgram of the pollutant per cubic meter of air [50]. For each census block, the individual lifetime cancer risk associated with each HAP is calculated by combining exposure concentration estimates with available UREs and inhalation reference concentrations. Cancer risks of different HAPs are assumed to be additive and are summed to estimate an aggregate lifetime cancer risk for each census block, measured in persons per million. This risk would be an excess cancer risk that is in addition to other cancer risks borne by a person not exposed to these HAPs. We assigned the 2005 NATA lifetime cancer risk estimates (from on-road mobile sources) from each census block in the Greater Houston MSA to all geocoded survey respondents who reside in the block.

#### 2.3.3. Explanatory Variables: Socio-Demographics and Hazard Experience

The second set of explanatory factors comprise socio-demographic variables from the structured survey that have been found to have a significant relationship with environmental risk perception in previous studies. A description of the survey variables used for this analysis is provided in Table 1. To examine the effect of race/ethnicity, the primary focus of our research, we included four dichotomous variables that represent mutually exclusive categories: non-Hispanic White, non-Hispanic Black, Hispanic/Latino origin (of any race), and other non-Hispanic minority.

In terms of control variables relevant to environmental risk perception, we included the gender (sex) and age (in years) of each survey respondent. The amplification of risk perception among females compared to males is well-documented [51,52,53,54]. Previous research has also demonstrated how gender modifies relationships between race and risk perception, especially in communities exposed to environmental hazards [23,24]. Several risk perception studies have found evidence of a ‘White male’ effect [23,24,25,26,27], which suggests that women and non-White men are more concerned about local environmental health risks than White men. Most studies have found that age correlates negatively with environmental concern, although there is growing evidence to suggest that this is a cohort effect where more recent generations tend to be more informed and concerned about environmental risks than previous ones [7,52,55]. 

To evaluate socioeconomic status (SES), another important dimension of risk perception, we used a continuous and standardized variable that combines 2011 annual household income levels with the level of education for the individual in the household with the highest attainment (Cronbach’s alpha = 0.67), using principal components analysis. The evidence on how SES influences environmental risk perception is somewhat mixed [7]. Individuals of higher SES can be expected to focus more energy and time on environmental issues than those who are less socioeconomically affluent [55,56]. Another body of research, however, challenges this assertion arguing that affluent individuals with high self-perceptions of agency and power are more likely to dismiss environmental concerns and risks, because they have more control in their daily lives [57,58]. In addition to SES, we included renter-occupant status as a control variable that represents housing tenure, or lack of home ownership. Similar variables have been used in prior studies as an indicator of housing instability, as well as lower levels of access to resources, political engagement, and involvement in local decision-making [16,59,60]. Finally, we included past experience with air pollution (based on whether the respondent or other household members suffered illnesses or health problems caused by exposure to air pollution) as an additional control variable. Perceived levels of personal risk have been found to be strongly and positively correlated with the frequency and intensity of people’s experience with various hazard events [61,62,63,64], including air pollution [57]. Consequently, we can expect survey respondents who have been negatively impacted by air pollution-related problems in the past to exhibit higher levels of health risk perception compared to other respondents in our sample.

Descriptive statistics for the entire set of variables used in our analysis are provided in Table 2, based on the original data. This table also indicates the number of valid observations for each survey variable in our data.

### 2.4. Statistical Methodology

We used generalized estimating equations (GEEs), a multivariate analysis technique appropriate for clustered data, to analyze determinants of air pollution health risk perception. Prior to running the GEEs, multiple imputation (MI) was employed to account for non-response bias associated with missing values for the control variables derived from our household survey. MI is considered to be the best practice for addressing missing data in statistical analysis [64,65,66] and it involves creating multiple sets of values for missing observations using a regression-based approach. MI is used to avoid the bias that can occur when missing values are not missing completely at random [67] and is particularly appropriate for self-reported survey data [68]. Using SPSS (version 22) software, 20 imputed datasets were specified to increase power and 200 between-imputation iterations were used to ensure that the resulting imputations were independent of each other [62]. To generate the results reported for our GEEs, SPSS was used to run the statistics 20 times (once per imputed dataset) and pool the results. 

Our multivariate analysis utilized GEEs with robust covariance estimates that extend the generalized linear model of Nelder and Wedderburn [69] to accommodate clustered data. GEEs have been used extensively for analyses of clustered observations in the biological and epidemiological sciences [70,71,72], as well as in recent EJ studies of the Greater Houston MSA [16,73]. However, to our knowledge, they have not been employed in environmental risk perception analysis based on survey data. GEEs are particularly appropriate for this study because they relax several assumptions of traditional regression models and impose no strict distributional assumptions for the variables analyzed, while accounting for geographic clustering of survey respondents in the study area.

To fit a GEE model, clusters of observations must be defined based on the assumption that observations from within a cluster are correlated, while observations from different clusters are independent. Our cluster definition was based on median year of housing construction, obtained from the 2010–2014 American Community Survey, by county of location, for census tracts in which surveyed households reside. Specifically, we defined clusters of census tracts based on median decade of housing construction (“2000 or later”, “1990 to 1999”, “1980 to 1989”, “1970 to 1979”, “1960 to 1969”, and “1950 to 1959”) by county (*n* = 8), which yielded a total of 48 different clusters. This cluster definition approach was selected over other alternatives such as census tract of residence, since GEE techniques assume dependence of observations within clusters and independence between clusters. These assumptions would be less reliable if only census tracts were utilized to define clusters, because data for households living in separate tracts, but within the same urban developmental context with similar socio-demographic characteristics, would be treated as independent [16]. The median year of home construction by county of cluster definition used here can be expected to closely correspond with the urban developmental context within which households are nested. A similar approach has been used in previous studies utilizing the GEE approach for household-level survey data [16,74].

GEEs also require the specification of an intracluster dependency correlation matrix, known as the working correlation matrix [70,71]. For the GEEs presented here, the working correlation matrix structure was specified as exchangeable, since this specification assumes constant intracluster dependency. Since our scaled dependent variable contained both negative and positive values, a linear specification (normal distribution with an identity link function) was employed for the GEE models.

## 3. Results

In order to explore the statistical effects of our explanatory variables on APHRP, three multivariate models based on the GEE approach are utilized. We used two different GEEs to examine the role of: (1) race/ethnicity; and (2) other independent variables/controls; separately and collectively. While the first model (model 1) only includes non-Hispanic Black and Hispanic status, the second model (model 2) includes these two racial/ethnic variables, cancer risks from exposure to industrial and vehicular air pollution, as well as the set of control variables representing socio-demographic characteristics. Model 3 encompasses all variables included in model 2, in conjunction with additional terms that represent two-way interactions between racial/ethnic and gender variables. Non-Hispanic White status was treated as the reference group in all models and thus excluded from the GEEs. The pooled GEE results, based on our multiply imputed dataset, are summarized in Table 3. The statistical significance of these independent variables is estimated on the basis of two-tailed *p*-values from the Wald’s chi-squared test. To check for multicollinearity, the multicollinearity condition index (MCI) was calculated for the combination of variables included in the multiple regression model. For all models, the MCI was found to be smaller than 30, indicating the absence of serious collinearity problems among the explanatory variables.

In Model 1, the positive coefficients for both non-Hispanic Black and Hispanic status indicate significantly higher levels of APHRP for respondents in these racial/ethnic groups compared to non-Hispanic Whites. After the inclusion of cancer risk and other control variables in model 2, non-Hispanic Black status continues to indicate statistically higher APHRP than non-Hispanic White status, but the coefficient for Hispanics becomes non-significant. While excess cancer risk from industrial air pollution shows a significantly positive association with APHRP, vehicular air pollution risk has a non-significant effect. In terms of our control variables, APHRP is found to be significantly higher among respondents who are female and those who have previously suffered from air pollution-related health problems. When gender-based interaction terms were added in model 3, variables that were significantly related to APHRP in model 2 (i.e., non-Hispanic Black, industrial air pollution risk, female, and past air pollution experience) retain their statistically significant associations. The interactions between non-Hispanic Black and female status, as well as Hispanic and female status are significant, which suggest that the relationship between air pollution risk perception and race/ethnicity is conditional on the gender of the survey respondent. When other explanatory variables are held constant or at zero, APHRP scores are found to be the highest for non-Hispanic Black males (0.246) and females (0.113), followed by Hispanic males (−0.109), White females (−0.128), and Hispanic females (−0.145). White male respondents indicate the lowest APHRP scores (−0.497), compared to the other combinations of race/ethnicity and gender. 

## 4. Discussion

This article sought to improve the current state of knowledge regarding the major factors shaping local perceptions of air pollution health risk by examining racial/ethnic differences in perceived risks, the spatial pattern of cancer risk from exposure to industrial and vehicular pollutants, and the role of specific socio-demographic characteristics in forming these perceptions. Our statistical results provide strong evidence of racial/ethnic disparities in air pollution risk perceptions in the Greater Houston MSA. Our multivariate analysis indicated that personal perceptions of air pollution health risk are significantly higher for both non-Hispanic Black and Hispanic residents, compared to non-Hispanic Whites and other non-Hispanic minorities when exposure to industrial and vehicular air pollutants and other socio-demographic factors are not considered. Although the differences in perceived health risk between non-Hispanic White and Hispanic respondents became non-significant, the White–Black disparity remained highly significant even after considering the effects of exposure to industrial/vehicular air pollution risks and other socio-demographic factors that shape risk perception. 

Since the 1980s, EJ researchers have documented how Black communities in this MSA and the city of Houston, in particular, are disproportionately exposed to industrial pollutants, hazardous waste, and other forms of environmental contamination [5,32,35,75,76]. The studies in Robert Bullard’s widely cited and highly influential book *Dumping in Dixie* [5] indicate that predominantly Black neighborhoods in Houston were deliberately targeted for facility siting decisions associated with municipal landfills, incinerators, and industrial facilities. Given this long history of toxic exposure, it is not surprising to find significantly greater awareness or concern regarding health risks from air pollution for non-Hispanic Black respondents in our survey, compared to other racial/ethnic groups. 

Risk perception levels were found to be significantly higher for those exposed to greater risk from industrial sources but not for those exposed to transportation sources of risk, after racial/ethnic status and other control variables were included in the multivariate model. Recent research on perceptions of air quality near busy highways also indicate that local residents often have a beneficial relationship with vehicles and roadways, which translates to lower health risk perception for traffic pollution, compared to industrial pollution [77]. For the Greater Houston MSA, our results suggest that local awareness regarding air pollution generated by the large petrochemical industries is likely to be substantially higher than public cognition of transportation-related emissions, a relatively recent and less conspicuous health hazard in this metropolitan area. 

It is also important to consider that several previous studies reported a disconnect between personal risk perceptions and scientifically-measured air pollution levels in Houston [36] and other U.S. urban areas [40,78,79]. This potential disparity between perception and measurement is often conceptualized as a ‘halo effect’ where individuals are reluctant to attribute high levels of air pollution to their own neighborhood or home metropolitan area [36,57]. While our study did not find any empirical support for a local halo effect in the Greater Houston MSA, the results are consistent with those of other risk perception studies which concluded that proximity to industry leads to higher awareness and concern for air pollution [80,81,82].

In terms of other characteristics, female respondents and those who have suffered health problems related to air pollution in the past indicated significantly higher levels of environmental risk perception, after controlling for other explanatory factors and interaction effects. These results do not differ from the findings of previous perception studies that report similar statistical relationships with perceived risk for gender [83] and previous hazard experience [84]. Our findings also elucidate that gender (sex) strongly influences the race-risk perception relationship. The significant interaction results support the “White male” hypothesis by indicating that non-Hispanic White men exhibit lower levels of air pollution risk perception compared to non-Hispanic White women and racial/ethnic minorities of both sexes. These results match the findings of risk perception studies that examined the “White male” effect in polluted environments [23,24,79], even though previous research did not specifically control for local health risks or exposure to industrial or vehicular pollutants. For both Non-Hispanic Blacks and Hispanics, men indicated significantly higher levels of perceived air pollution risk than women. Perceived risk levels for both Black men and women, however, were higher than those of non-Hispanic Whites and Hispanics of both sexes. This finding again suggests that Black residents of the Greater Houston area, regardless of gender, view the outcomes of air pollution to be racially determined and reflective of discriminatory historical trends that have been placed various environmental hazards and pollution sources in their communities. 

Additional data and further research are needed to develop a more comprehensive understanding on how race/ethnicity and local exposure to air pollution shape public perceptions of environmental health risk. Firstly, our dependent variable provides a general assessment of air pollution health risk perception for local residents, but does not incorporate emission sources or categories. Future research could focus on formulating and utilizing variables that examine personal risk perceptions associated with specific air pollution sources such as oil refineries, petrochemical plants, automobile exhaust, construction, and/or agricultural dust in the local area. Secondly, our measurement of household-level air pollution risk focuses only on cancer risk and does not include non-cancer health effects such as respiratory or neurological risks that are also associated with ambient exposure to industrial and vehicular air pollutants. It is important to explore the use of additional data sources that provide geographically detailed information on other air pollution-related health risks and adverse health outcomes that can be linked to the surveyed households. Thirdly, the racial/ethnic categories used in our analysis (i.e., Black or Hispanic status) are somewhat broad and imply a degree of homogeneity that may not exist with minority populations in this metropolitan area. While our study contributes to the growing trend of looking beyond White/non-White dichotomies in the literature on race/ethnicity and perceived environmental risks by including Hispanics [7,85], recent research has emphasized that treating Hispanics as single group could be problematic because this group encompasses people of different cultural, economic, and social characteristics, as well as migration histories and countries of origin [60,86]. Given the diversity of the Black or Hispanic population in larger urban areas such as Greater Houston, future analyses of risk perceptions and race/ethnicity should attempt to disaggregate these broad categories based on social class and other contextually relevant characteristics.

## 5. Conclusions

In summary, our study demonstrates significantly higher concerns regarding air pollution health risk for non-Hispanic Black residents of Greater Houston, even after accounting for their exposure to industrial and vehicular air pollutants, other socio-demographic factors, and the effects of spatial clustering in our survey data. The fact that our statistical controls were insufficient in explaining risk perception differences between White and Black respondents confirms themes that environmental justice advocates and scholars have repeatedly asserted—environmental health risks in U.S. urban areas are unequally distributed and this form of inequality is particularly palpable for residents who live near industrial pollution sources, as well as highways and roadways [7]. This research affirms that movements, programs, and policy interventions that seek to reduce disproportionate environmental health burdens need to include the voices and address the needs of local residents who themselves perceive the greatest levels of environmental risk.

## Figures and Tables

**Table 1:** Description of survey variables and related metrics.

Variable	Survey Question	Metric
Air pollution health risk perception (APHRP)	Two-item factor based on: (1) How much of a problem do you think air pollution is in this urban area (scale: 1–5)? (2) How concerned are you about the possibility of air pollution causing health problems to you, or members of your household (scale: 1–5)?	1 = not a problem at all; 5 = a very serious problem. 1 = not concerned; 5 = extremely concerned.
Non-Hispanic White	Which of the following best describes your race: White (if not Hispanic)?	1 = yes; 0 = no
Non-Hispanic Black	Which of the following best describes your race: Black or African American (if not Hispanic)?	1 = yes; 0 = no
Hispanic	Are you of Hispanic, Latino, or Spanish origin?	1 = yes; 0 = no
Other non-Hispanic minority	Which of the following best describing your race: Asian/ American Indian/other race (if not Hispanic)?	1 = yes; 0 = no
Female	Are you female?	1 = yes; 0 = no
Age of respondent	Based on: In what year were you born?	Continuous
Renter status	Is this home rented?	1 = yes; 0 = no
Socioeconomic status	Two-item factor based on: (1) What was your total household income for the year 2011 before taxes? (2) Thinking about the person in your household with the highest educational degree received or level of school completed—what is the highest grade/level of school this person has completed?	1 = < $10,000 2 = $10,000–19,999 3 = $20,000–29,999 4 = $30,000–39,999 5 = $40,000–49,999 6 = $50,000–74,999 7 = $75,000–99,999 8 = $100,000–149,999 9 = $150,000–249,999 10 = > $249,999 0 = No formal schooling–21 = Ph.D. degree
Past experience	Have you or other members of your household ever suffered from illnesses or health problems that you believe were caused or worsened by exposure to air pollution?	1 = yes; 0 = no

**Table 2:** Summary statistics for analyzed variables, Greater Houston MSA.

	N	Min	Max	Mean	SD
***Dependent Variable*:**					
Air pollution health risk perception (APHRP)	586	−2.21	1.44	0.02	0.99
***Quantitative Independent Variables*:**					
Industrial air pollution risk ^a^	586	0.00	157.00	6.88	14.26
Vehicular air pollution risk ^a^	586	0.00	27.06	4.49	4.62
Age of respondent	566	18.00	94.00	56.28	15.41
Socioeconomic status (SES)	452	−3.10	2.31	0.02	1.00
***Dichotomous Independent Variables*:**		**Yes (1)**	**No (0)**	**Proportion of Values Coded “1”**	
Non-Hispanic White	579	284	295	0.49	
Non-Hispanic Black	577	110	467	0.19	
Hispanic	581	122	459	0.21	
Other non-Hispanic minority	573	57	516	0.10	
Female	585	380	205	0.65	
Renter status	568	108	460	0.19	
Past air pollution experience	586	193	393	0.33	

^a^ Excess lifetime cancer risk estimated to result from continuous exposure to a pollutant at a concentration of 1 μg/m^3^ in air.

**Table 3:** Pooled results (beta estimates) from generalized estimated equations predicting APHRP.

Variables	Model 1	Model 2	Model 3
Non-Hispanic White	Ref.	Ref.	Ref.
Non-Hispanic Black	0.528 **	0.387 **	0.743 **
Hispanic	0.218 *	0.118	0.388
Other non-Hispanic minority	−0.214	−0.164	−0.247
Industrial air pollution risk		0.004 **	0.003 **
Vehicular air pollution risk		0.005	0.005
Female		0.227 *	0.369 **
Age of respondent		−0.002	−0.001
Renter status		−0.044	−0.021
Socioeconomic status		−0.069	−0.061
Past air pollution experience		0.714 **	0.720 **
Non-Hispanic Black X Female			−0.502 *
Hispanic X Female			−0.405 **
Other non-Hispanic minority X Female			0.258
Intercept	−0.104	−0.371	−0.497 **
(Scale)	0.928	0.786	0.775
*Model fit: (QIC)*	539.4–552.4	464.2–473.1	457.9–468.2
*Multicollinearity Index*	2.409	15.298	27.387

* *p* < 0.05; ** *p* < 0.01; QIC: Quasi-likelihood under the independence model criterio.
